# A novel nomogram to predict the local tumor progression after microwave ablation in patients with early‐stage hepatocellular carcinoma: A tool in prediction of successful ablation

**DOI:** 10.1002/cam4.2606

**Published:** 2019-11-12

**Authors:** Chao An, Songsong Wu, Zhimei Huang, Jiayan Ni, Mengxuan Zuo, Yangkui Gu, Tianqi Zhang, Jinhua Huang

**Affiliations:** ^1^ State Key Laboratory of Oncology in South China Department of Minimal Invasive Intervention Collaborative Innovation Center for Cancer Medicine Sun Yat‐sen University Cancer Center Guangzhou China; ^2^ Department of Ultrasonography Fujian Provincial Hospital Shengli Clinical Medical College of Fujian Medical University Fuzhou PR China

**Keywords:** ablation techniques, hepatocellular carcinoma, neoplasm staging, nomogram, recurrence

## Abstract

**Objectives:**

To develop a nomogram for the local tumor progression (LTP) in patients with early‐stage hepatocellular carcinoma (HCC) after computed tomography‐guided percutaneous microwave ablation (CT‐PMWA) and to assess clinical‐pathologic risk factors for individual LTP estimation. Furthermore, we compared the prognostic predictive ability for LTP between the nomogram and the traditional staging systems.

**Methods:**

This retrospective study was approved by the institutional review board. Five hundred and forty treatment‐naïve patients with HCC according to the Milan criteria, who subsequently underwent CT‐PMWA were reviewed from 2009 to 2019. Baseline characteristics were collected to identify the risk factors for the determination of LTP after CT‐PMWA. The multivariate Cox proportional‐hazards model based on significant prognostic factors of LTP was used to construct the nomogram, which was then assessed for its predictive accuracy using mainly the Harrell's C‐index and time‐dependent area under the curve (tAUC).

**Results:**

After a median follow‐up time of 28.7 months, 6.5% (35/540) patients had LTP. The nomogram was developed based on the tumor size, tumor number, Child‐Turcotte‐Pugh (CTP) grade, platelet, and alanine aminotransferase (ALT). The nomogram had good calibration and discriminatory abilities in the training set, with C‐indexes of 0.799 (95% confidence interval (CI): 0.738, 0.860), and tAUCs of 0.844 (CI: 0.728, 0.895), that were greater than those of traditional staging systems. Internal validation with 1000 bootstrap resamples had a good C‐index of 0.735 (CI: 0.648, 0.816).

**Conclusions:**

The nomogram model can be used to predict accurately LTP after CT‐PMWA for early‐stage HCC, as well as to assist physicians during the therapeutic decision‐making process.

## Graphical abstract

Local tumor progression (LTP) is a key criterion for evaluating the technical success of various thermal ablation techniques. Insufficient ablation margin is an independent risk factor associated with LTP, however, it is difficult to measure accurately. This normogram demonstrated higher predictive accuracy compared with traditional staging systems and may prove to be useful in centers that do not have the facilities for measuring ablation margin.

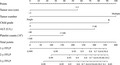

**Key Points**

- Local tumor progression is a key criterion for evaluating the technical success of various thermal ablation techniques.
- Insufficient ablation margin is an independent risk factor associated with local tumor progression, however, it is difficult to measure accurately.
- This normogram demonstrated higher predictive accuracy compared with traditional staging systems and may prove to be useful in centers that do not have the facilities for measuring ablation margin.

## 1 INTRODUCTION

As a first‐line treatment option to early‐stage hepatocellular carcinoma (HCC) according to the American National Comprehensive Cancer Network (NCCN) guidelines, microwave ablation (MWA) has been widely used with conspicuous advantage, including higher intratumoral temperature, less operation time, and dependence the electrical conductivities.^1^, ^2^, ^3^ With the recent advances in MWA (eg, hydrodissection techniques, three‐dimensional preoperative planning, multi‐modal image fusion navigation, and thermal monitoring needle), this safe and effective treatment can achieve satisfactory survival outcomes.^4^, ^5^, ^6^

Local tumor progression (LTP) is one of the most important criteria for evaluating the technical success of thermal ablation techniques.^7^ The independent risk factors associated with LTP have been reported in previous studies,^8^, ^9^, ^10^, ^11^ which mainly including insufficient ablation margin (AM), tumor size, and challenging locations (eg, subcapsular and perivascular). Because of the limited assessment ability with side‐by‐side routine axial images by radiologists, although AM has been a good prognostic indicator for LTP, the accurate judgment is difficult to obtain easily. In addition, high‐power MWA was less affected by “heat‐sink” effect when the nodule locates abutting major vessels, due to intratumoral temperature rise rapidly. Therefore, the risk factors of LTP after MWA of HCC remain controversial.

Over the last decade, several studies have reported that LTP rate after various of thermal ablation methods in HCC, which ranged between 5.1% and 20.7%.^12^, ^13^, ^14^, ^15^, ^16^ However, there is a lack of some studies that comprehensively assesses LTP risk factors. In order to ensure the high success rate of MWA, accurate risk prediction methods urgently need to be developed. Nomograms derived from hazard functions are straightforward graphic tools that have been applied to predict oncological outcomes after patients with HCC who undergo radiofrequency ablation (RFA) with more discriminatory abilities compared with traditional staging systems,^17^, ^18^ but are reported less frequently for the prediction of LTP after MWA.

Here, we conducted this study to construct a nomogram and to independently validate this scoring system for LTP after percutaneous MWA of early‐stage HCC. Furthermore, we compared the prognostic predictive ability of this nomogram for estimating LTP risk score of per‐patient after MWA with that of traditional staging systems.

## 2 MATERIALS AND METHODS

### 2.1 Patients and treatments

**Figure 1:**
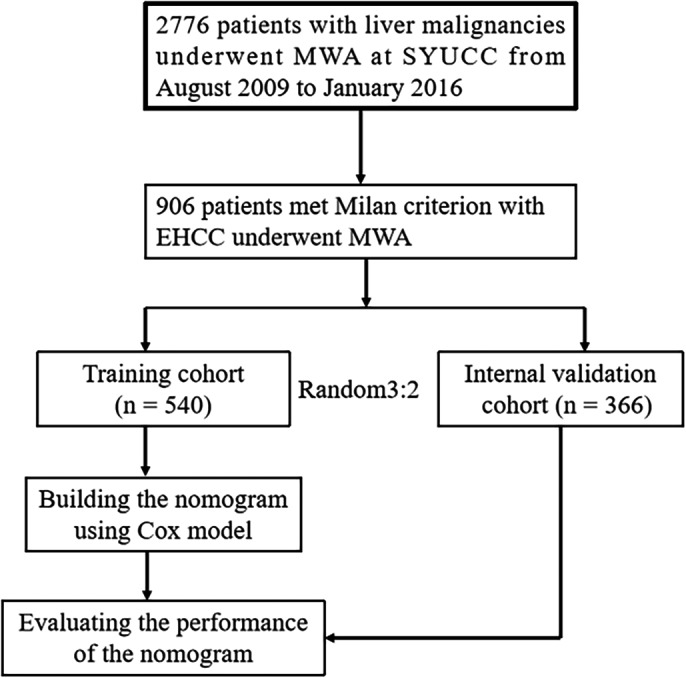
Flow diagram shows study patient accrual process

The protocol for this single‐center, retrospective study was approved by the Ethics Committee at Sun Yat‐sen University Cancer Center (Guangzhou, China), with the need to obtain informed consent was waived. Strict adherence to the principles of the Declaration of Helsinki was upheld. Our cohort consisted of 906 treatment‐naïve patients (180 females, 726 males; average age 57.4 ± 10.8 years) with 1390 HCC lesions (mean diameter, 2.5 ± 1.0 cm) which were diagnosed based on the Milan criteria. All patients underwent computed tomography‐guided percutaneous microwave ablation (CT‐PMWA). Their electronic medical records from August 2009 to January 2016 were reviewed for this study. HCC was diagnosed based on the European Association for the Study of Liver and the American Association for the Study of Liver Disease guidelines.^19^ Newly diagnosed HCC cases were deliberated at multidisciplinary meetings which comprised of oncologists, surgeons, pathologists, hepatologists, and radiologists (JHH and WJF, with 25 and 25 years of experience) in order to decide on the best course of management for each patient. Inclusion criteria for this study included following parameters: (a) patients with Child‐Turcotte‐Pugh (CTP) grade A or B (Eastern Cooperative Oncology Group (ECOG) performance status 0); (b) a single tumor maximum size <5 cm and tumor number <3; (c) no extrahepatic metastasis or major vascular incursion; and (d) HCC lesions that were surgically unresectable or patients who voluntarily accepted MWA treatment. Those patients underwent other treatments before MWA, and those with severe coagulopathy (ie, platelet count <50 cells × 10^9^/L, prothrombin activity <40% and prothrombin time >25 seconds) were excluded. Figure 1 demonstrates the exclusion and inclusion criteria as well as the patient enrollment pathways. MWA procedure protocol and the device itself were documented in previous report.^20^ Interventional radiologists (JHH, FJZ, and ZMH with 25, 25, and 8 years of experience in performing MWA, respectively) were responsible for performing all percutaneous ablative procedures.

### 2.2 Data analysis

Patient assignment into the training and validation datasets was done via computer‐generated randomization based on 3:2 ratio, the reason of grouping is to ensure adequate sample number in the training dataset. Ultimately, there was a total of 366 patient in the validation dataset and 540 patients in the training dataset. Extracted demographic and clinical information are as follows: (a) demographic and history (sex, age, comorbidities (ie, hypertension, diabetes, heart disease, renal disease, and esophageal gastric varices), etiology, cirrhosis and CTP grade, and α‐fetoprotein [AFP] level); (b) tumor features (maximal diameter, number, and location abutting major vessels which was defined as direct HCC contact with either the main hepatic veins, inferior vena cava, secondary portal vein branches, or any vessel with a diameter <3 mm); (c) ablation parameters (ablation frequency, number of antenna, number of insertion, ablation time, ablation power, and ablation session); (d) laboratory findings (serum albumin, serum total bilirubin, platelet counts, international normalized ratio (INR), aspartate aminotransferase [AST] and alanine aminotransferase [ALT]); and (e) HCC stage (tumor‐node‐metastasis [TNM] stage, Barcelona clinic liver cancer [BCLC] stage, China staging [CS], and Okuda score).

### 2.3 Assessment and follow‐up

Contrast‐enhanced multiphase images (magnetic resonance imaging [MRI] or computed tomography [CT]) were performed 3 days after the last course of a defined ablation protocol, in order to assess the efficacy of treatment. Technique effectiveness was defined as comprehensive local necrosis 1 month following the treatment.^21^ In cases where adequate ablation was achieved, serum AFP as well as contrast‐enhanced CT or MRI were performed again at 1 and 3 months after CT‐PMWA and subsequently at intervals between 3 and 6 months. For patients with suspected metastasis a chest CT, bone scan, or positron emission tomography‐CT was performed. Local tumor progression (LTP) was defined the appearance of tumor foci at the edge of the ablation zone after at least one contrast‐enhanced follow‐up study had documented adequate ablation and there was an absence of viable tissue in the target tumor and the surrounding AM assessed using imaging criteria. LTP results based on a large sample were showed in previous studies (Table 1). Subsequent treatment for recurrent HCC depended on the patient preferences and the clinical practices of radiologists. Patients who experienced recurrence were generally managed via surgical resection, MWA, RFA, TACE, and systemic chemotherapy, depending on the general condition, hepatic function, and the tumor location.

**Table 1:** The studies related to local tumor progression after microwave ablation of hepatocellular carcinoma

Study group	No. of tumors	Mean tumor size (cm)	Complete ablation (%)	LTP (%)	Follow‐up (mo)
Liang et al	477	3.8 ± 1.8	—	8	31.4
Yu et al	928	2.8 ± 1.4	—	8.6	20.3
Poggi et al	194	2.7	94.3	5.1	19.5
Zhai et al	221	4.0	90.9	15.7	41
Zheng et al	220	3.0 ± 2.0	92.8	7.2	25.2
Dong et al	71	3.7 ± 0.5	87.5	16.7	16.8
Musa et al	26	5.7 ± 0.7	73.1	19.2	26.4
Xu et al	82	5.6 ± 0.3	89.0	20.7	9.5

Abbreviation: LTP, Local tumor progression.

### 2.4 Statistical analysis

The primary study endpoint was the emergence of LTP post‐MWA. Baseline clinical parameters that were compared between training and validation sets were selected based on the 2001 European Association for the Study of the Liver guidelines. Categorical variables were subjected to Pearson's χ^2^ analysis or Fisher's exact tests, while continuous variable was subjected to the Mann‐Whitney *U* test. The Kaplan‐Meier method with log‐rank test was used to evaluate the rates of LTP. Univariate and multivariate analyses of independent LTP factors were evaluated through the forward stepwise Cox regression model. Cox model‐derived β coefficients were applied for normogram construction in order to assess the relationship between LTP and selected variables. Scores for each patient in the validation group were calculated based on an established normogram during internal normogram validation.

Three modalities were used to assess the discriminative ability of the models. First, calibration was done by plotting the predicted probability of LTP at 12, 24, and 36 months vs observed probability. These time points were derived based on a study from YJ et al who established the median LTP occurrences in patients with liver malignancies who received first‐line MWA.^9^ The training set provided the regression coefficients that were used to calculate risk scores. Second, the time‐dependent area under the curve (tAUC) was calculated using the training cohort. Using this method, we were able to achieve more comprehensive data regarding the predictive power of the model in contrast to the c‐index method, as the tAUC involves computing sensitivity and specificity. Third, a decision curve analysis (DCA) was carried out to establish and contrast the clinical value between the nomogram model and traditional staging systems through calculation of the net benefits at each risk threshold probability. The net benefit was ascertained by subtracting the proportion of all false‐positive values from the proportion of true‐positive values and weighted against the relative harm caused by forgoing treatment in contrast to the negative impact of unnecessary treatments. In addition, the Wald test, LR test, and Akaike information criterion (AIC) were compared across different statistical models, which were generated using parametric analysis. Statistical analysis was undertaken using the SPSS 21.0 (SPSS) program and the RMS package of the R software version 3.5.1 (http://www.r-project.org/). All tests of significance were two‐sided and a *P* value <0.05 was interpreted to carry statistical significance.

## 3 RESULTS

### 3.1 Baseline characteristics and intermediate‐term LTP

**Figure 2:**
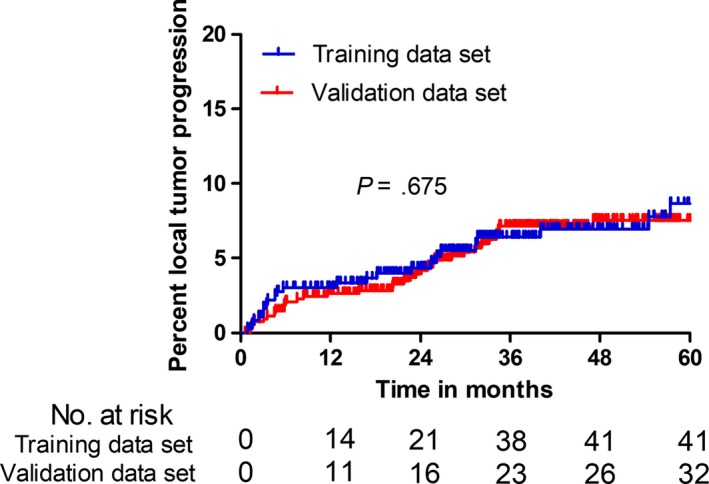
Kaplan‐Meier local tumor progression curves of comparison between the training dataset and the validation dataset

A total of 906 patients with 1390 HCC nodules who underwent CT‐PMWA over a total of 7 years were reviewed in this study. There were 894 patients with 1362 lesions that received treatment using a 2450 MHz microwave generator and with the rest receiving treatment using a 915 MHz microwave generator. There was no statistically significant difference between the 100% of technique effectiveness rate between the training data and validation dataset (*P* = 1.000). Patients were followed up for a median of 28.7 months (range, 7.6‐110.5 months) and 35 patients (6.5%) had experienced a confirmed LTP in the training dataset. The overall LTP rates at 1, 2, 3, 4, and 5 years were 2.6%, 4.2%, 7.1%, 7.5%, and 7.5%, respectively, in the training dataset and 3.0%, 4.3%, 6.4%, 7.0%, and 8.6%, respectively, in the validation dataset, showing no significant statistical difference (*P* = .675) (Figure 2). There were no significant differences between clinical characteristics and follow‐up data between both of the groups (*P* = .764‐0.787) (Table 2).

**Table 2:** Baseline patient characteristics

Variables	Training set (n = 540)	Validation set (n = 366)	*P* value
Demographic and history			
Mean age ± SD (y) (range)	57.2 ± 10.7 (24‐83)	57.6 ± 10.9 (25‐81)	.764^a^
Sex			.139^b^
Male	424 (78.5)	302 (82.5)	
Female	116 (21.5)	64 (17.5)	
Mean BMI ± SD (kg/m^2^) (range)	22.5 ± 5.2 (21.4‐25.8)	21.8 ± 6.4 (20.9‐24.3)	.527^a^
Performance status			.898^b^
0	513 (95.0)	347 (94.7)	
1	27 (5.0)	19 (5.3)	
Comorbidities			.519^b^
Absence	79 (14.6)	48 (13.1)	
Presence	461 (85.4)	318 (86.9)	
Etiology			.333^b^
HBV	423 (78.3)	287 (78.4)	
HCV	48 (8.9)	42 (11.5)	
Alcohol‐induced	9 (1.7)	7 (1.9)	
Other	60 (11.1)	30 (8.2)	
Cirrhosis			.484^b^
Absence	50 (9.3)	29 (7.9)	
Presence	490 (90.7)	337 (92.1)	
CTP grade			.648^b^
A	516 (100)	352 (97.6)	
B	24 (0)	14 (2.4)	
Median AFP level (ng/mL) (range)	234.6 (3.2‐1381.2)	221.9 (4.8‐762.8)	.254^a^
Tumor data			
Mean maximal tumor diameter ± SD (cm) (range)	2.5 ± 0.9 (0.9‐5.0)	2.6 ± 1.0 (0.8‐5.0)	.188^a^
No. of tumors	812	578	.139^b^
Single	316 (78.8)	196 (58.8)	
Multiple	224 (21.2)	170 (41.2)	
Abutting major vessel			.445^b^
Absence	79 (39.6)	47 (40.3)	
Presence	461 (60.4)	319 (59.7)	
Treatment parameter			
Ablation frequency (MHz)			.801^b^
915	4 (95.0)	2 (95.0)	
2450	536 (95.0)	364 (95.0)	
Mean No. of antenna ± SD (range)	1.7 ± 0.6 (1‐2)	1.6 ± 0.4 (1‐2)	.782^a^
Median No. of insertion (range)	2.6 (1‐4)	2.8 (1‐6)	.092^a^
Median ablation time ± SD (minutes) (range)	9.4 (2.2‐47.7)	11.2 (3.3‐50.3)	.362^a^
Mean ablation power ± SD (Watts) (range)	54.8 ± 10.8 (45‐70)	55.6 ± 9.4 (45‐60)	.712^a^
Ablation sessions^c^^,^ d			.578^b^
1	656 (80.8)	460 (81.6)	
2	156 (19.2)	118 (18.4)	
Laboratory findings			
Mean albumin level ± SD (g/L) (range)	35.1 ± 11.3 (16.1‐45.7)	35.4 ± 10.7 (11.6‐46.4)	.492^a^
Median total bilirubin level (μmol/L) (range)	19.3 (2.2‐62.9)	18.4 (3.7‐62.1)	.971^a^
Median ALT (U/L) (range)	34.2 (8.1‐182.5)	35.6 (9.6‐212.3)	.681^a^
Median AST (U/L) (range)	34.9 (12.9‐199.3)	35.7 (8.8‐187.6)	.219^a^
Median platelet counts (×10^9^) (range)	115.5 (67.2‐178.2)	108 (55.3‐166.7)	.562^a^
Mean INR ± SD (range)	1.13 ± 0.21 (0.87‐1.38)	1.15 ± 0.32 (0.89‐1.49)	.898^a^
HCC stage			
BCLC stage			.388^b^
0	180 (33.3)	112 (30.6)	
A	360 (66.7)	254 (60.4)	
TNM stage			.139^b^
I	316 (78.8)	196 (58.8)	
II	224 (21.2)	170 (41.2)	
CS stage			.091^b^
Ia	302 (53.5)	172 (47.0)	
Ib	220 (38.9)	169 (46.2)	
II	43 (7.6)	25 (6.8)	
Okuda score			.355^b^
I	172 (31.9)	106 (29.0)	
II	368 (68.1)	260 (71.0)	
Technique effectiveness^c^^,^ d	812/812 (100)	578/578 (100)	1.000^b^
Complications^c^	5/540 (0.9)	3/366 (0.8)	.867^b^
Follow‐up (y)			.787^a^
Median	30.3	27.0	
Range	0.6‐110.5	3.2‐109.3	

Note

Except where indicated, data are numbers of patients. Data in parentheses are percentages and were calculated by using the total number of patients in each group as the denominator. SD = standard deviation. *P* < .05 indicated a significant difference.

Abbreviations: AFP:α‐fetoprotein; ALT, alanine aminotransferase; AST, aspartate aminotransferase; BCLC, barcelona clinic liver cancer; BMI, body mass index; CS, China staging; CTP, Child‐Turcotte‐Pugh; HBV, hepatitis B virus; HCV, hepatitis C virus; INR, international normalized ratio; TNM, tumor‐node‐metastasis.

a Student's *t* test.

b Pearson's χ^2^ test.

c Data in parentheses are percentages.

d Data are the number of treatments.

### 3.2 Construction of the nomogram for LTP

**Figure 3:**
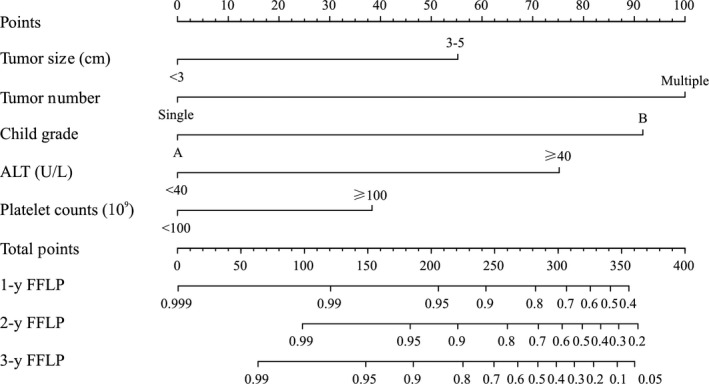
The nomogram was developed in the validation dataset, with tumor size, tumor number, ALT level, CTP grade, and platelet level

Eighteen possible risk factors (including sex, age, comorbidities, etiology, cirrhosis, CTP grade, BCLC grade, AFP, tumor size, number, location abutting major vessels, serum total bilirubin, serum albumin, AST, ALT, INR, platelet, and sessions) for LTP were evaluated by univariate and multivariate analysis. The following five variables were discovered to be strongly related to the occurrence of LTP after MWA in the training dataset, including tumor size, tumor number, CTP grade, ALT, and BCLC grade (Table 3). In the multivariate analyses, larger tumor size (3‐5 cm) (*P* = .002), number of nodules (two and three nodules) (*P* < .001), CTP grade B (*P* = .001), lower platelet levels (<100 × 10^9^) (*P* = .040), and higher ALT level (>40 IU/L) (*P* = .017), were independent risk factors linked to LTP (Table 3). A nomogram for predicting the LTP after MWA in training dataset, which is shown in Figure 3. The five preidentified prognostic risk factors were used to derive the normogram, with each component allocated a predetermined score. The predicted risk of LTP at 1‐, 2‐, and 3‐year post‐MWA was the sum of points for each patient.

**Table 3:** Factors associated with poor LTP after MWA for HCC according to univariate and multivariate analysis

Factors	No. of patients	Univariate analysis multivariate analysis			
		HR (95% CI)	*P* value	HR (95% CI)	*P* value*
Age (y)		1.908 (0.948, 3.838)	.070	—	—
<65	412				
≥65	128				
Gender		0.839 (0.462, 1.524)	.565	—	—
Male	424				
Female	116				
Comorbidities		2.129 (0.651, 6.961)	.211	—	—
Absence	79				
Presence	461				
Etiology				—	—
HBV	423	—	.516		
HCV	48	1.482 (0.592, 3.712)	.400		
Alcohol‐induced	9	0.682 (0.163, 2.859)	.601		
Other	60	0.000 (0.000, 1.141)	.975		
Cirrhosis		1.404 (0.508, 3.875)	.513	—	—
Absence	50				
Presence	490				
Tumor size (cm)		2.072 (1.250, 3.436)	.005	2.887 (1.456, 5.727)	.002
<3	390				
3‐5	150				
Tumor number		5.627 (2.992, 10.581)	<.001	6.816 (3.033, 15.320)	<.001
Single	316				
Multiple	224				
Abutting major vessels		1.018 (0.501, 2.068)	.960	—	—
Absence	461				
Presence	79				
AFP (ng/mL)		1.227 (0.907, 1.661)	.185	—	—
≤20	373				
>20	167				
Albumin (g/L)		1.710 (0.756, 3.866)	.197	—	—
<35	171				
≥35	369				
Total bilirubin (μmol/L)		0.719 (0.334, 1.549)	.400	—	—
<20.5	365				
≥20.5	175				
ALT (U/L)		0.278 (0.085, 0.907)	.034	0.236 (0.072, 0.775)	.017
<40	404				
≥40	136				
AST (U/L)		1.345 (0.743, 2.434)	.328	—	—
<40	421				
≥40	119				
Platelet count (×10^9^)		1.428 (0.732, 2.788)	.296	2.086 (1.033, 4.214)	.040
<100	285				
≥100	255				
INR		1.012 (0.582, 1.760)	.967	—	—
<1.1	316				
≥1.1	224				
Ablation session		2.148 (0.783, 3.603)	.349	—	—
1	536				
>1	4				
CTP grade		3.110 (1.097, 8.817)	.033	5.813 (1.966, 17.188)	.001
A	516				
B	24				
BCLC grade		8.356 (1.144, 61.053)	.036	—	—
0	180				
A	360				

Note

Data in parentheses are 95% confidence intervals.

Abbreviations: AFP, α‐fetoprotein; ALT, alanine aminotransferase; AST, aspartate aminotransferase; BCLC, barcelona clinic liver cancer; CTP, Child‐Turcotte‐Pugh; HBV, hepatitis B virus; HCC, hepatocellular carcinoma; HCV, hepatitis C virus; HR, hazard ratio; CI, confidence intervals; INR, international normalized ratio; MWA, microwave ablation.

* *P* values were determined with Cox proportional hazards regression models. *P* < .05 indicated a significant difference.

### 3.3 Validation of the nomogram

**Figure 4:**
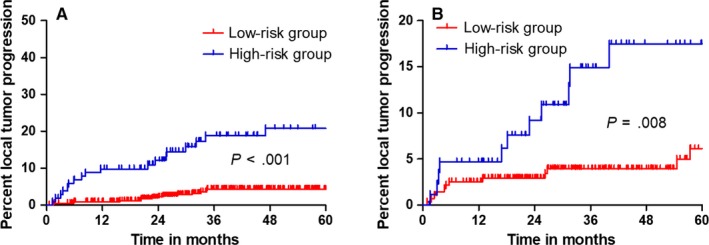
Graphs show results of Kaplan‐Meier local tumor progression (LTP) analyses according to the nomogram in the training dataset (A) and those in the validation dataset (B). A significant association of the nomogram with the LTP was shown in the training dataset, which was then confirmed in the validation dataset

**Figure 5:**
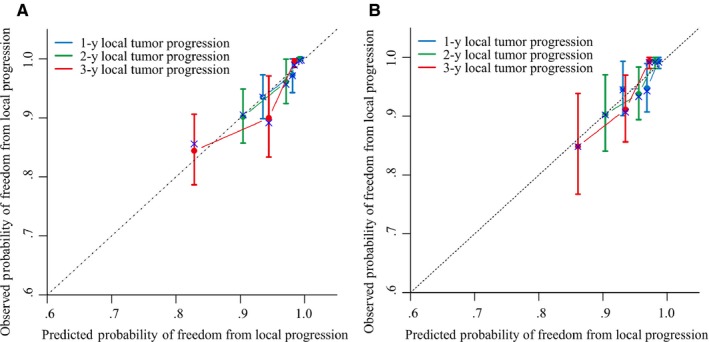
Calibration curve for predicting local tumor progression (LTP) after MWA at (A) 1, 3, and 5 years in the training dataset and at (B) 1, 3, and 5 years in the validation dataset. Nomogram‐predicted probability of LTP is plotted on the x‐axis; actual LTP is plotted on the y‐axis

The LTP rate stratified by the risk score of nomogram was then used to plot Kaplan‐Meier curves in the validation and training datasets (Figure 4). The training dataset demonstrated strong associations between the normogram and LTP occurrence (*P* < .001; HR = 0.088, 95% confidence interval [CI]: 0.037, 0.208), a finding that was replicated in the validation dataset (*P* = .008; HR = 0.278, 95% CI: 0.107, 0.715). The LTP rates in both low‐ and high‐risk groups for both validation and training sets are depicted in Table 4. LTP prediction was 0.799 (95% CI: 0.738, 0.860) in the training dataset when assessed using the C‐index. The likelihood of 1‐, 2‐, and 3‐year LTP was consistent between clinical observation and normogram prediction (Figure 5A). The C‐index for LTP prediction was 0.732 (95% CI: 0.648, 0.816) in the validation dataset. The calibration plot for the likelihood of 1‐, 2‐, and 3‐year LTP demonstrated an optimal agreement between clinical observation and normogram prediction, a finding that coincided with the internal validation dataset (Figure 5B).

**Table 4:** Local tumor progression rate in high‐risk and low‐risk groups

Parameter	Training dataset			Validation dataset		
	High‐risk group	Low‐risk group	Total	High‐risk group	Low‐risk group	Total
No. of patients	103	437	540	89	277	366
No. of LTP	19	16	35	12	14	26
At 1 y	9.8%	0.9%	2.6%	4.7%	2.5%	3.0%
At 2 y	12.1%	2.3%	4.2%	9.2%	2.9%	4.3%
At 3 y	18.8%	4.3%	7.1%	14.9%	3.9%	6.4%
At 4 y	20.8%	4.3%	7.5%	17.5%	3.9%	7.0%
At 5 y	20.8%	4.3%	7.5%	17.5%	6.1%	8.6%

Abbreviation: LTP, local tumor progression.

### 3.4 Discrimination ability of the nomogram

C‐index, Wald test, LR test, and AIC estimates for the various conventional staging systems such as the tumor‐node‐metastasis (TNM) staging system, the Barcelona Clinic Liver Cancer (BCLC) grade, the China stage (CS) grade, the Okuda score, and the clinical‐pathologic normogram, are depicted in Table 5. Of these scoring systems, the normogram demonstrated the best discrimination capability as well as the highest C‐index in predicting LTP after MWA. These differences in C‐indices between different prognostic methods were of statistical significance (*P* < .05, for each comparison). Among all of the classifiers or models, the normogram demonstrated the highest concordance probability (0.799) and the lowest AIC (381.48).

**Table 5:** Performance of models

Model	C‐index	95% CI	*P* value*	Wald test	LR test	AIC
Nomogram in TS	0.799	0.738, 0.860	1.000	38.97	44.05	381.48
Nomogram in VS	0.732	0.648, 0.816	.647	32.11	23.78	393.32
BCLC stage	0.597	0.512, 0.625	<.001	4.38	9.11	408.43
TNM stage	0.715	0.632, 0.735	.032	15.82	19.47	398.06
CS stage	0.597	0.523, 0.613	<.001	6.63	5.85	411.68
Okuda score	0.602	0.550, 0.645	.001	5.37	5.05	412.48

Abbreviations: AIC, Akaike information criterion; BCLC, Barcelona clinic liver cancer; CS, China staging; TNM, tumor‐node‐metastasis; TS, training set; VS, validation set.

* Other models compared with nomogram in training set.

**Figure 6:**
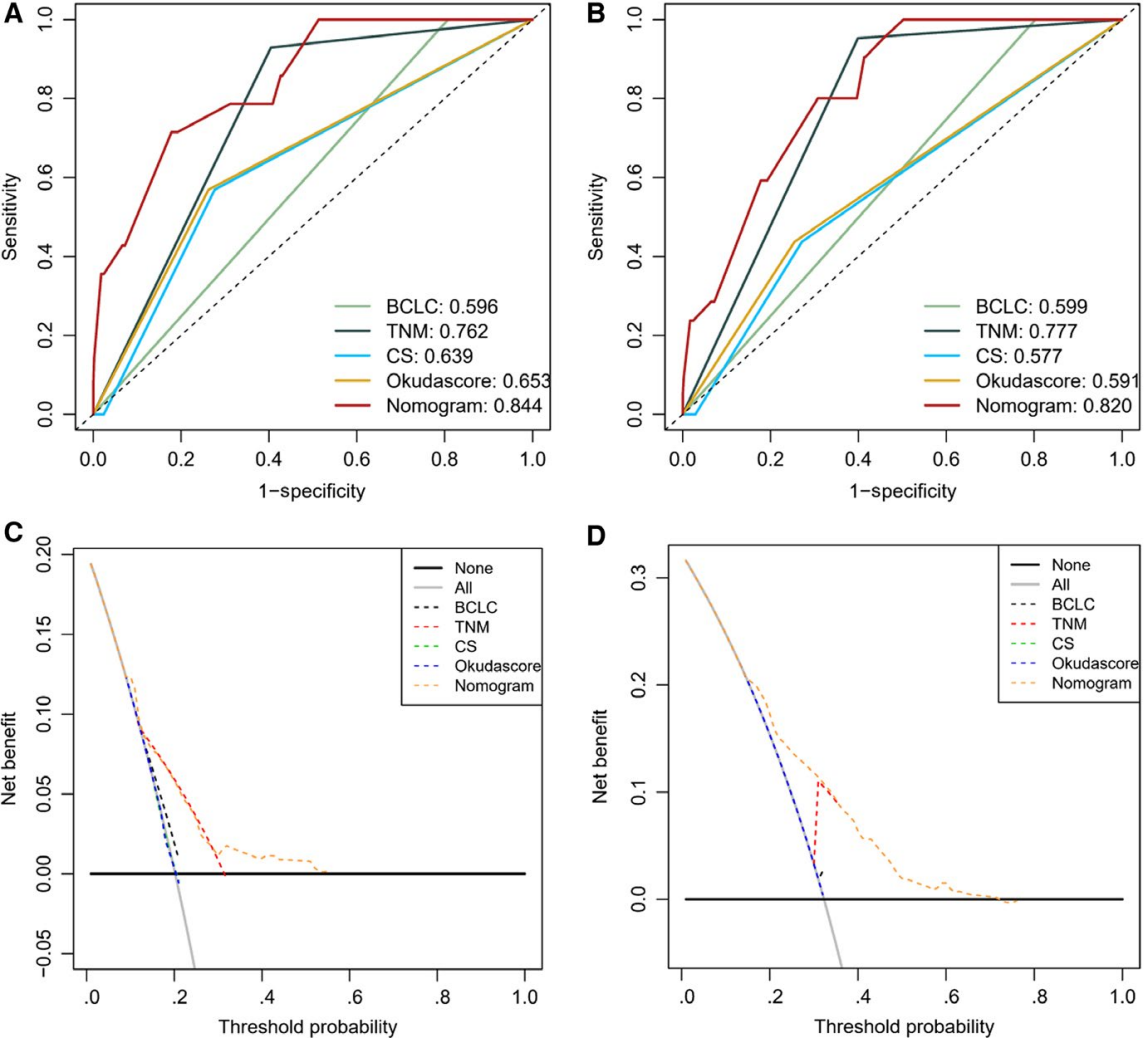
Comparison of predictive accuracy for local tumor progression after MWA between the nomogram and the conventional system. (A) AUC analyzed in training dataset; (B) AUC analyzed in validation dataset. Decision curve analysis (DCA) for each model. The y‐axis measures the net benefit. The net benefit was calculated by summing the benefits (true‐positive results) and subtracting the harms (false‐positive results). (A) DCA analyzed in training dataset; (B) DCA analyzed in validation dataset

The tAUC of the normogram was used to assess its discriminative ability. tAUC in the training dataset was 0.844 (95% CI: 0.728, 0.895) and 0.820 (95% CI: 0.733, 0.865) in the validation dataset (Figure 6A,B). These scores were noted to be higher than those in other traditional staging system models. A DCA demonstrates that using a normogram was able to confer higher benefits in contrast to conventional staging systems across the majority of the range of reasonable threshold probabilities (Figure 6C,D).

## 4 DISCUSSION

A certain proportion of patients with unresectable HCC has been found to benefit from MWA. Successful ablation requires an adequate tumor‐free margin (5‐10 mm) and LTP is the most ideal assessment in technical effectiveness.^22^, ^23^, ^24^ Currently, traditional staging systems have been developed to classify patients with HCC. Nevertheless, there are several defects regarding the traditional staging system as follow: first, the current staging system is purely based on the anatomical extent of the disease, staging systems do not completely reflect the biological heterogeneity of HCC patients; second, other risk factors are not taken into account in current staging systems.^25^, ^26^, ^27^, ^28^ The risk prediction ability of these systems to LTP of an individual after MWA was suboptimal. Therefore, we develop a novel predictive modeling system using the nomogram methodology.

In our study, the total incidence of LTP was 6.5% per patient after MWA during the median follow‐up period of 28.7 months. Compared with other studies,^2^, ^9^, ^29^ the present study achieved relatively optimistic LTP results in a large cohort with long‐term follow‐up. By reviewing the medical records of patients accept MWA as the primary treatment for HCC in early stage, based on five independent prognostic risk factors (tumor size, tumor number, ALT level, CTP grade, and platelet level) of pre‐MWA patients, a nomogram tailored to the individual patient with the ability to predict the LTP was developed. Also, we tested the available risk model for LTP in a large, contemporary population of patients treated by MWA in the internal validation dataset. The nomogram for the LTP after MWA had C‐indexes of 0.799, and tAUCs of 0.844, which is more exact and reliable than the widely used traditional staging systems. This new model can also be used to select the patients for inclusion in clinical trials on the basis of their postablation LTP risk, whereas randomization can be stratified using either a two‐ or three‐risk grouping. Although the usefulness of the proposed nomogram lacked external validation, the DCA and AUC demonstrated that the nomogram was superior to the clinical staging system across the majority of the range of reasonable threshold probabilities, which in the meanwhile indicated that the nomogram added incremental value to the traditional staging system and other clinical‐pathologic risk factors for individualized estimation.

Most studies have shown that the perivascular tumors were significantly associated with the LTP after RFA,^30^, ^31^, ^32^, ^33^ however, it was not a parameter in the nomogram in our study. This result suggests that it is related to the characteristics of MWA less affected by “heat‐sink” effect. We are expecting further prospective randomized studies to confirm our conclusions. Moreover, for tumor size (range, 2‐5 cm), the risk of LTP is also increasing step by step followed to per 1 centimeter increasing (Figure S1). In early‐stage HCC, the size is a critical risk factor for LTP in the patients underwent MWA. Also, tumor number is a non‐negligible indicator and multiple nodules increased the difficulty of physicians performing ablation. Strikingly, many reports believe that insufficient ablation margin (AM) is an important risk factor for LTP after thermal ablation. As AM measurement is not routinely available in the majority of centers and the methodology is not globally standardized, interestingly, according to our findings. The nomogram developed without AM also showed greater predictive accuracy compared with the current staging system. This outcome indicated that the nomogram was still useful for the centers that do not have AM measurement available. Compared with the long‐term outcome OS, LTP is an end point that avoids extended follow‐up and enables earlier adjustment of therapy. Thus, our study may present a more efficient tool that enables earlier personalized treatment.

Several limitations associated with the present study, including, first, the relatively small sample size, the retrospective nature of data collection, and the lack of external validation, our preferred design should be a prospective cohort study, where there is full control for ensuring that no bias is introduced for all relevant risk factors; second, due to time span is too large, the LTP rate may be affected by the learning and mastery of MWA; and third, several vital factors including AM were not incorporated into the nomogram and these data fail to acquire on all patients in our cohort. Although a large‐scale independent prospective multicenter validation cohort is warranted to assess the generalizability of the reported findings, the DCA and AUC used in this study, which enables the assessment of clinical relevance without the requirement for additional validation data in a traditional decision analytic approach.

In conclusion, the nomogram has the potential to be used for risk stratification for LTP in patients with early‐stage HCC after MWA. Moreover, the nomogram may serve as a potential tool to guide individual postablation care for those patients, although this will require further external validation before widespread implementation in clinical practice.

## ETHICS APPROVAL AND CONSENT TO PARTICIPATE

The ethics committee board of the Sun Yat‐sen University Cancer Center, approved the use of patients with early HCC after MWA for this study.

## CONFLICT OF INTEREST

The authors declare no conflict of interest.

## AUTHOR CONTRIBUTIONS

CA and ZMH were involved in conceptualization. ZMH was involved in methodology. TQZ was involved in software and investigation. SSW, CA, and JYN were involved in validation. YKG was involved in formal analysis. SSW was involved in resources. MXZ was involved in data curation. CA was involved in writing—original draft preparation. JHH was involved in writing—review and editing and funding acquisition. Guarantor name, Jinhua Huang.

## Data Availability

Please contact the corresponding author for all data requests.
